# Extra-Neurological Characterization of Seckel Syndrome-Model Mice Harboring *CEP152* Variants

**DOI:** 10.3390/cells15131148

**Published:** 2026-06-24

**Authors:** Nanako Hamada, Koki Ichihashi, Tohru Matsuki, Ikuko Iwamoto, Atsuo Nakayama, Akira Hara, Koh-ichi Nagata

**Affiliations:** 1 Department of Molecular Neurobiology, Institute for Developmental Research, Aichi Developmental Disability Center, Kasugai 480-0392, Japan; nhamada@inst-hsc.jp (N.H.); iwamoto@inst-hsc.jp (I.I.); 2 Department of Tumor Pathology, Gifu University School of Medicine, Gifu 501-1194, Japan; koki.i0905@gmail.com (K.I.); hara.akira.y7@f.gifu-u.ac.jp (A.H.); 3 Department of Cellular Pathology, Institute for Developmental Research, Aichi Developmental Disability Center, Kasugai 480-0392, Japan; matsukit@inst-hsc.jp (T.M.); atsuon@inst-hsc.jp (A.N.); 4 Department of Neurochemistry, Nagoya University Graduate School of Medicine, Nagoya 466-8550, Japan; 5 Hirano General Hospital, Gifu 501-1192, Japan

**Keywords:** CEP152, Seckel syndrome, testis development, macrocytic anemia, mouse models, pathogenic variants

## Abstract

**Highlights:**

**What are the main findings?**

- Two *Cep152* Seckel syndrome mouse models show extra-neurological defects, including impaired spermatogenesis with mitotic abnormalities and increased apoptosis in spermatogonia.
- Both models exhibit hematological changes consistent with macrocytic anemia, and the analysis also reveals reduced oligodendrocyte-lineage readouts in one model (e.g., fewer Olig2-positive cells and decreased Opalin expression).

**What are the implications of the main findings?**

- CEP152 dysfunction impacts proliferative progenitor populations beyond the brain, providing a mechanistic basis for multi-organ manifestations in Seckel syndrome.
- Comparable extra-neurological phenotypes across two variant models suggest tissue-dependent modulation/compensation, highlighting the need to evaluate *CEP152*-related disease as a systemic developmental disorder rather than a purely neurodevelopmental condition.

**Abstract:**

Centrosomal protein 152 (CEP152) is a key regulator of centriole architecture and function, essential for proper cell division and polarity. Pathogenic variants in *CEP152* cause Seckel syndrome (SCKL), a systemic disorder characterized by microcephalic primordial dwarfism. However, the mechanisms underlying its multi-organ manifestations remain poorly understood. To investigate this, we utilized two mouse models harboring patient-derived *CEP152* variants, *Cep152*^W105*/K897*^ and *Cep152*^Q32P/Q32P^. While our previous work focused on neurodevelopmental defects, here we systematically analyzed extra-neuronal phenotypes. We identified impaired spermatogenesis, characterized by defective mitosis and increased apoptosis in spermatogonia, as well as hematological abnormalities indicative of macrocytic anemia. In addition, we found reduced expression of *Opalin*, a gene involved in oligodendrocyte differentiation, and decreased numbers of Olig2-positive oligodendrocytes, suggesting broader glial deficits beyond recently characterized neuronal abnormalities. Collectively, our results highlight the role of CEP152 dysfunction in multi-systemic abnormalities of SCKL and provide an integrative view of its impact on both neuronal and extra-neuronal development.

## 1. Introduction

The centrosome, the primary microtubule-organizing center in animal cells, consists of a pair of centrioles surrounded by a protein-rich matrix known as the pericentriolar material. This organelle plays a central role in various cellular processes, including cell division, establishment of cell polarity, and cellular motility [1,2,3]. Increasing evidence has highlighted that genetic variants affecting centriolar and centrosomal proteins are implicated in a spectrum of developmental disorders, particularly those characterized by microcephaly and dwarfism [4,5,6,7,8].

Centrosomal protein 152 (CEP152), encoded by the *CEP152* gene, was initially identified as a mammalian centrosomal component through proteomic analyses [9,10]. It is the mammalian ortholog of the *asterless* gene in *Drosophila*, which is well-established as an essential regulator of cell division and tissue development [11,12]. Subsequent studies have elucidated the crucial role of CEP152 in centriole biogenesis and centrosome function, primarily serving as a scaffold for procentriole assembly [13,14,15,16]. CEP152 exerts its function through interactions with several key proteins, including CEP63, CPAP/CENPJ/SAS-4, CEP192, CDK5RAP2/CEP215, WDR62, and PLK4 [13,15,17,18,19,20]. Biochemical mapping has shown that the C-terminal region of CEP152 is essential for binding CEP63, CPAP, and CEP192 [13,19,20,21], while the N-terminal region mediates interaction with PLK4 [22,23].

Pathogenic variants in *CEP152* and its interacting partners have been linked to autosomal recessive primary microcephaly, a rare neurogenic disorder characterized by mitotic abnormalities and reduced brain size, and to Seckel syndrome (SCKL, OMIM: 210600). SCKL belongs to the group of microcephalic primordial dwarfism syndromes, which are rare, autosomal recessive, single-gene disorders presenting with intrauterine and postnatal growth retardation. Clinically, SCKL manifests with proportionate short stature, distinctive facial dysmorphisms, microcephaly, and varying degrees of intellectual disability [24,25].

In this study, we sought to elucidate the multi-systemic consequences of CEP152 dysfunction by utilizing two previously established *Cep152* mouse models: *Cep152*^W105*/K897*^, mimicking compound heterozygous variants (c.314G > A, p.W105* and c.2689A > T, p.K897*), and *Cep152*^Q32P/Q32P^, carrying a homozygous variant (c.95A > C, p.Q32P). While our previous work focused primarily on the neurodevelopmental abnormalities observed in these models, here we extend our investigation to extra-neuronal tissues, specifically the testes and hematopoietic system, in line with the systemic nature of SCKL. Furthermore, we revisit the neural tissues to explore potential glial involvement, aiming to provide a more comprehensive view of *CEP152*-related pathology. Through integrative morphological, cellular, and molecular analyses, we aim to shed light on how *CEP152* variants contribute to both neuronal and extra-neuronal manifestations of SCKL.

## 2. Materials and Methods

### 2.1. Ethics Statement

All animal experiments and related activities in this study complied with institutional guidelines and relevant national regulations under the jurisdiction of the Ministry of Education, Culture, Sports, Science, and Technology, Japan. Experimental protocols for animal care and use were reviewed and approved by the Animal Care and Use Committee of the Institute for Developmental Research, Aichi Developmental Disability Center (Approval Number: 2024-002, approved on 22 November 2024).

### 2.2. Animals

The generation of *Cep152*^W105*/K897*^ and *Cep152*^Q32P/Q32P^ mouse strains were described previously [26]. Mice were housed (one animal in each cage) with 12-to-12 h-light–dark cycle, humidity (60 ± 5%), with access to food and water ad libitum in individually ventilated cages. A total of 106 animals were used in this study. No samples or animals were excluded from the analysis. Animals were allocated to groups to balance sex and litter, minimizing subjective bias.

### 2.3. Antibodies

The rabbit polyclonal anti-activated cleaved Caspase3 (Casp3; Cell Signaling, Danvers, MA, USA, Cat# 9664, 1:400) and anti-phospho-Histone H3 (Ser10) (pHH3) (Cell Signaling, Cat# 9701, 1:400) were used. Mouse monoclonal anti-γ-tubulin (Sigma Aldrich, St. Louis, MO, USA, Cat# T6557, 1:1000) and rat monoclonal CD56 antibody (Leica Microsystems, Wetzlar, Germany, PA0191, 1:500) were also employed. As secondary antibodies, Alexa Fluor 488-, 568- and 647-labeled IgG (Abcam, Cambridge, UK, Cat# ab150077, Cat# ab175471, Cat# ab150075, respectively, at 1:1000 dilution) were used. 4′, 6-diamidino-2-phenylindole (DAPI) (Sigma-Aldrich, Cat# D9542, 0.2 μg/mL) was used to stain DNA.

### 2.4. Immunohistochemistry

*Cep152*^W105*/K897*^ and *Cep152*^Q32P/Q32P^ knock-in mice were generated as described previously [26]. Mice were deeply anesthetized using a combination of medetomidine (0.75 mg/kg), butorphanol (5 mg/kg), and midazolam (4 mg/kg) [27], followed by perfusion with 4% paraformaldehyde. Tissue samples were sectioned at a thickness of 100 μm using a vibrating microtome (VT1000, Leica Microsystems). Sections were blocked for 1 h in phosphate-buffered saline (PBS) containing 0.5% Triton X-100 and 0.1% bovine serum albumin, then incubated overnight at 4 °C with primary antibodies diluted in PBST (PBS with 0.05% Triton X-100). The next day, sections were incubated with secondary antibodies in PBST for 1 h, followed by DAPI staining for nuclear visualization. After 3 washes with PBST, sections were mounted with anti-fade mounting medium (PERMAFLUOR, Cat#TA-030-FM, Thermo Scientific, Waltham, MO, USA). Fluorescence images were acquired using a confocal laser scanning microscope (LSM-880, Carl Zeiss, Jena, Germany).

### 2.5. Reverse Transcription (RT)-PCR

Total RNA was extracted from hemispheres of adult wild-type and *Cep152*^W105*/K897*^ mice at postnatal day 60 (P60) using Sepasol^®^-RNA I Super (Nacalai Tesque Inc., Tokyo, Japan, Cat# G09379-97). The qPCR template was prepared using ReverTra Ace qPCR RT Master Mix with gDNA Remover (Toyobo, Osaka, Japan, Cat# FSQ-301). RT-PCR analysis was performed using THUNDERBIRD Next SYBR qPCR Mix (Toyobo, Cat# QPX-201) and a CFX96 Touch Real-Time PCR Detection System (Bio-Rad, Hercules, CA, USA), with the following primer sets: *Cnp* (forward: CGCCCACTCATCATGAACAC, reverse: TGGCTTCTCCTTTGCTCCTG); *Mag* (forward: GCTACAACCAGTACACCTTCTC, reverse: CCATACAACTGACCTCCACTTC); *Opa* (forward: CCTTGATCCAGCGAAGAAGAA, reverse: ACCGCCTAGGATTCTCAGATA); *Qdpr* (forward: AGCTCCTGGACACCCTTAGA, reverse: TTAGGCTTCCTGAGTTTGGC); *Trf* (forward: CCCTCTGTGACCTGTGTATTG, reverse: CTTTCTCAACGAGACACCTGAA). Relative mRNA expression levels were normalized to Gapdh and expressed relative to the corresponding WT control value, which was set to 1.0.

### 2.6. Statistical Analyses

For all cell imaging experiments, cell counting and trace analysis were performed by a staff member blinded to the experimental conditions. Statistical analyses were conducted using GraphPad Prism version 10.6.1 (GraphPad Software Inc., San Diego, CA, USA). Results are presented as mean ± SD. For comparisons between two groups, Welch’s *t*-test was used. For comparisons involving more than two groups, one-way analysis of variance (ANOVA) was performed, followed by Tukey–Kramer test for multiple comparisons. Statistical significance was defined as *p* < 0.05. Data normality was not formally assessed in this study, and no formal test for outliers was conducted. No data were excluded from the analyses. Box and whisker plots represent the median (horizontal bars), the 25th to 75th percentiles (box edges), and the minimum and maximum observed values (whiskers). The cross inside the boxes indicates the mean.

## 3. Results

### 3.1. Growth Defects in Cep152^W105*/K897*^ and Cep152^Q32P/Q32P^ Mice

**Figure 1:**
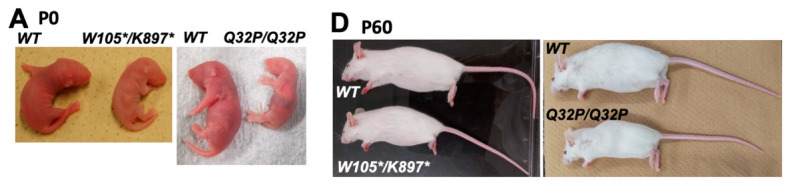

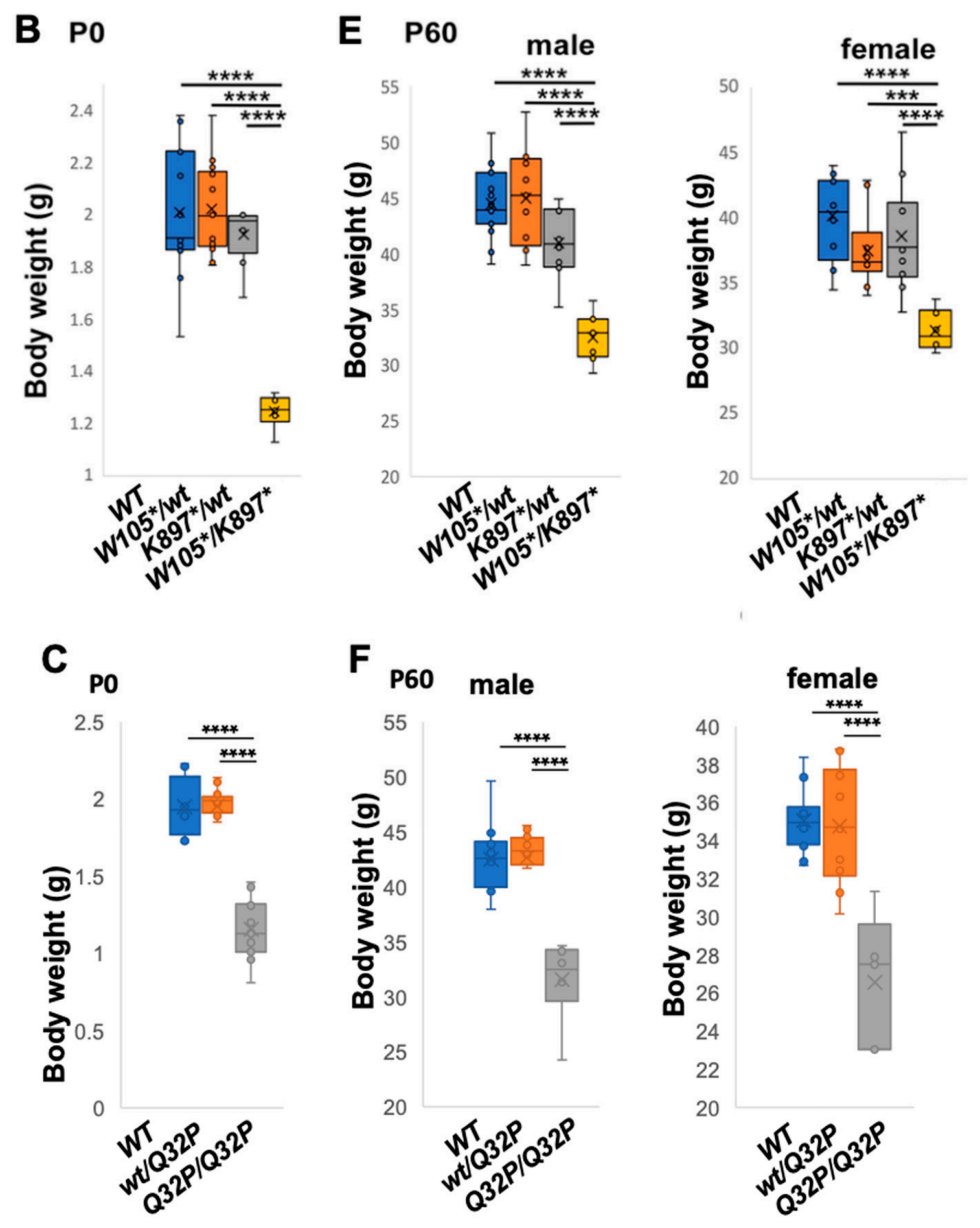
Growth defects in *Cep152*^W105*/K897*^ and *Cep152*^Q32P/Q32P^ mice. (**A**) Representative images of WT, *Cep152*^W105*/K897*^, and *Cep152*^Q32P/Q32P^ mice at P0. (**B**) Quantification of body weight at P0 for WT, *Cep152*^W105*/wt^, *Cep152*^K897*/wt^, and *Cep152*^W105*/K897*^ mice (*n* = 8, 13, 18, and 6, respectively). (**C**) Quantification of body weight at P0 for WT, *Cep152*^wt/Q32P^, and *Cep152*^Q32P/Q32P^ mice (*n* = 8, 6, and 15, respectively). (**D**) Representative images of WT, *Cep152*^W105*/K897*^, and *Cep152*^Q32P/Q32P^ mice at P60. (**E**) Quantification of body weight at P60 for WT, *Cep152*^W105*/wt^, *Cep152*^K897*/wt^, and *Cep152*^W105*/K897*^ mice. Sample sizes are as follows. Male: WT (*n* = 19), *Cep152*^W105*/wt^ (*n* = 13), *Cep152*^K897*/wt^ (*n* = 8), *Cep152*^W105*/K897*^ (*n* = 9); Female: WT (*n* = 12), *Cep152*^W105*/wt^ (*n* = 10), *Cep152*^K897*/wt^ (*n* = 10), *Cep152*^W105*/K897*^ (*n* = 8). (**F**) Quantification of body weight at P60 for WT, *Cep152*^wt/Q32P^, and *Cep152*^Q32P/Q32P^ mice. Sample sizes are as follows. Male: WT (*n* = 14), *Cep152*^wt/Q32P^ (*n* = 12), *Cep152*^Q32P/Q32P^ (*n* = 6); Female: WT (*n* = 12), *Cep152*^wt/Q32P^ (*n* = 10), *Cep152*^Q32P/Q32P^ (*n* = 5). Statistical analyses were performed as described in Section 2. For details of the box plots, see the same section. *** *p* < 0.001, **** *p* < 0.0001.

To evaluate the systemic growth phenotypes associated with *CEP152* variants, we analyzed body weight progression in both compound heterozygous (*Cep152*^W105*/K897*^) and homozygous (*Cep152*^Q32P/Q32P^) mutant mouse models. As early as P0, both mutant lines exhibited a significant reduction in average body weight compared to wild-type littermates (Figure 1A–C). This growth retardation phenotype persisted throughout development, with body weights remaining markedly lower at P60 in both male and female mice (Figure 1D–F). Notably, although *Cep152*^Q32P/Q32P^ mice exhibited more severe microcephaly than *Cep152*^W105*/K897*^ mice at P60 [26], their body weight at this stage did not significantly differ (Figure 1B,C,E,F). This suggests that both variants contribute similarly to postnatal growth impairment despite differing neurological severity. We further assessed whether heterozygous carriers exhibited similar growth abnormalities. Body weight measurements in *Cep152*^W105*/wt^, *Cep152*^K897*/wt^, and *Cep152*^Q32P/wt^ mice revealed no significant differences compared to wild-type controls at both P0 and P60 (Figure 1B,C,E,F), indicating that heterozygous loss of CEP152 function may not affect growth. This observation is consistent with the autosomal recessive inheritance pattern observed in SCKL.

### 3.2. Testicular Hypoplasia and Impaired Spermatogenesis in Cep152^W105*/K897*^ and Cep152^Q32P/Q32P^ Mice

**Figure 2:**
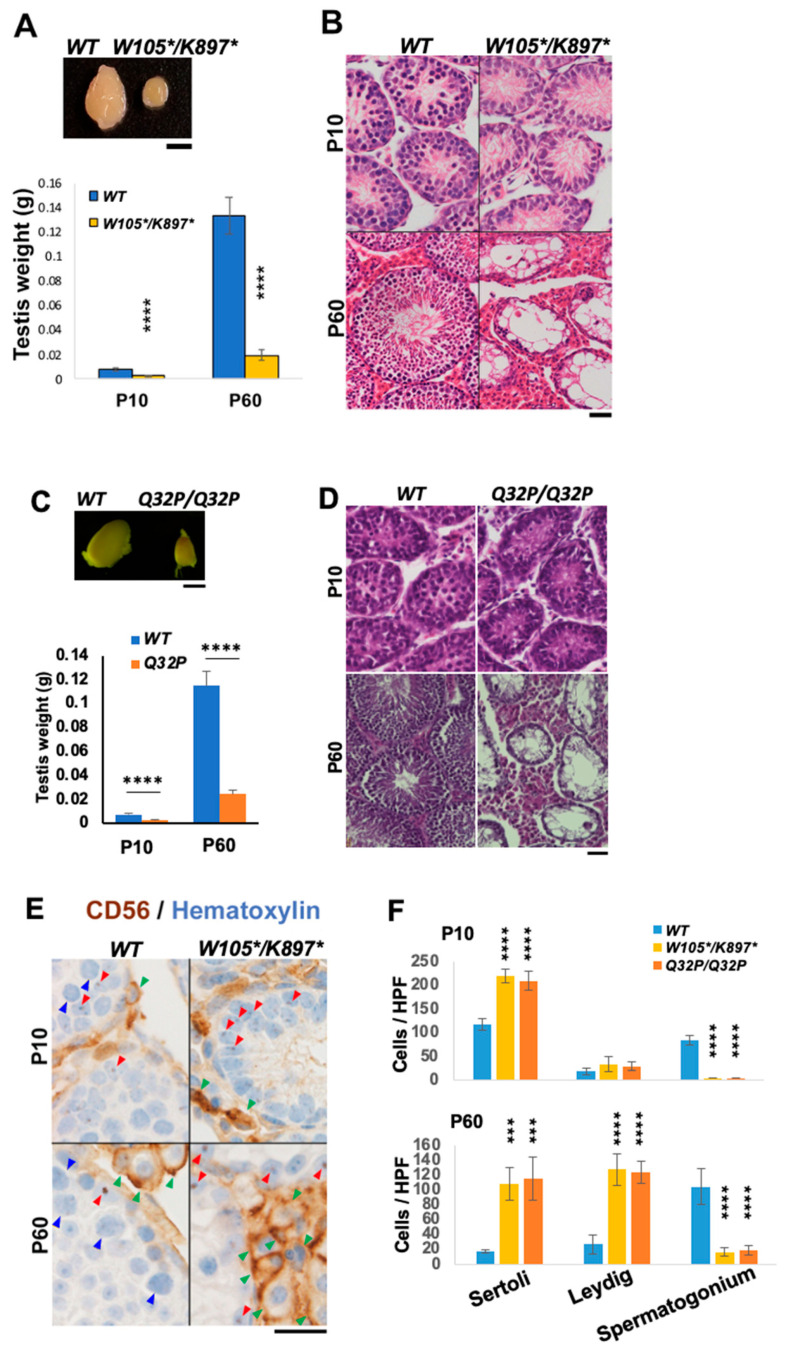
Defects in testis development in Cep152^W105*/K897*^ and Cep152^Q32P/Q32P^ mice. (**A**) Gross images of dissected testes of wild type (WT) and *Cep152*^W105*/K897*^ mice at P60. Graphs show mean testicular weights of the indicated genotypes at P10 (*n* = 7 for WT, and 5 for *Cep152*^W105*/K897*^) and P60 (*n* = 12 for WT, and 11 for *Cep152*^W105*/K897*^). (**B**) Histological examination of testes of *Cep152*^W105*/K897*^ at P10 or P60. Testes were dissected, paraffin-embedded, sectioned (4 μm), and double-stained with hematoxylin and eosin. Images were captured using a BZ-9000 microscope (Keyence Inc., Osaka Japan). (**C**) Gross images of dissected testes of WT and *Cep152*^Q32P/Q32P^ mice at p60. Graphs show mean testicular weights of the indicated genotypes at P10 (*n* = 5 for each genotype) and P60 (*n* = 9 for WT, and 5 for *Cep152*^Q32P/Q32P^). (**D**) Histological examination of testes of *Cep152*^Q32P/Q32P^ mice at P10 or P60. Analyses were done as in (**B**). (**E**) Double-staining of testes from WT and *Cep152*^W105*/K897*^ mice (P10 and P60) with hematoxylin (blue) and anti-CD56 (brown). Blue *arrowheads*, red *arrowheads*, and green *arrowheads* indicate spermatogonia, Sertoli cells, and Leydig cells, respectively. Note that CD56 is positive for cell membrane of Leydig cells and nucleoli of Sertoli cells. (**F**) Quantification analysis. Numbers of Sertoli cells, Leydig cells, and spermatogonia per high-power field (HPF; 400× magnification) from *Cep152*^W105*/K897*^ (**E**) and *Cep152*^Q32P/Q32P^ mice were scored at P10 and P60 (*n* = 4 for each genotype). *** *p* < 0.001, **** *p* < 0.0001. Scale bars; 2 mm (**A**,**C**), 30 μm (**B**,**D**), 20 μm (**E**).

Given that hypoplastic external genitalia, including cryptorchidism, are frequently observed in patients with SCKL (https://www.malacards.org/card/seckel_syndrome_1, accessed on 6 May 2025), we assessed whether similar reproductive abnormalities are present in *Cep152*^W105*/K897*^ and *Cep152*^Q32P/Q32P^ mice. Gross anatomical examination revealed a marked reduction in testis size in *Cep152*^W105*/K897*^ mice compared to wild-type littermates at P10 and P60 (Figure 2A). Histological analysis at P10 showed that, while the number and size of seminiferous tubules were comparable between mutants and controls, germ cell density was significantly reduced in *Cep152*^W105*/K897*^ testes (Figure 2B, upper panels). By P60, drastic reductions in the tubule size and cell density were observed (Figure 2B, lower panels). Similar findings were noted in *Cep152*^Q32P/Q32P^ testes (Figure 2C,D). To further characterize testicular abnormalities, we performed immunohistochemical staining for CD56, a marker for Leydig and Sertoli cells. CD56 staining marks Leydig cell membranes and Sertoli cell nucleoli. Both mutant lines showed a marked decrease in spermatogonia, accompanied by a relative increase in Leydig and Sertoli cells at P60 (Figure 2E,F). These results suggest impaired spermatogenesis and germ cell loss in these mutant mice.

**Figure 3:**
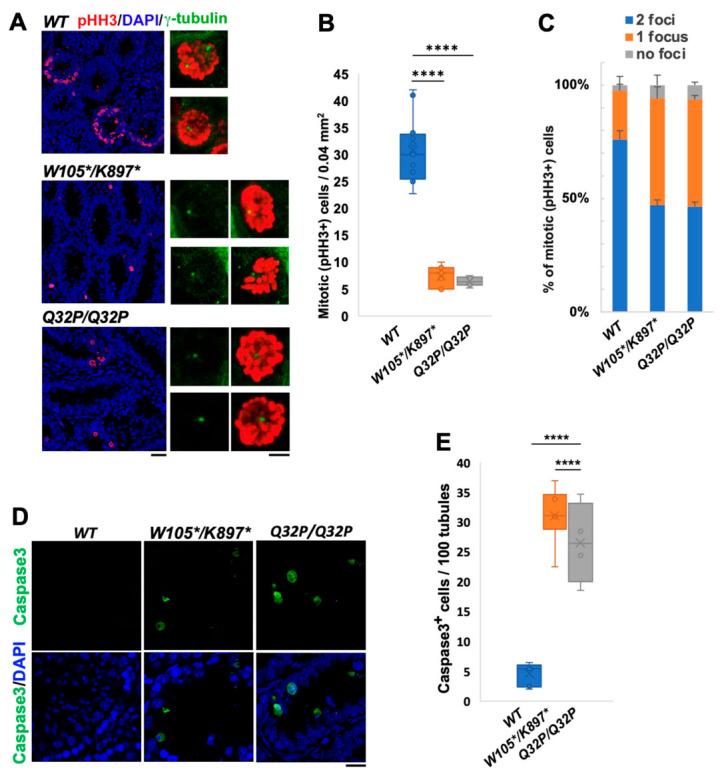
Analysis of mitotic spindle organization and apoptosis in spermatogonial progenitors of *Cep152*^W105*/K897*^ and *Cep152*^Q32P/Q32P^ mice. (**A**) Representative images of mitotic spermatogonia from WT, *Cep152*^W105*/K897*^, and *Cep152*^Q32P/Q32P^ mice at P10. Slices were triple-stained with anti-phospho-histone H3 (pHH3) (red), anti-γ-tubulin (green), and 4′,6-diamidino-2-phenylindole (DAPI) (blue). (**B**,**C**) Quantification of mitotic cells in (**A**). *n* = 4 animals for WT, 5 for *Cep152*^W105*/K897*^, and 5 for *Cep152*^Q32P/Q32P^; 50 cells per genotype scored. (**B**) Box-and-whisker plots indicate the number of pHH3-positive cells per 0.04 mm^2^ area. (**C**) The number of γ-tubulin foci in mitotic cells was scored and data are presented as mean + SD of individual testes. (**D**) Representative images of Casp3 (green)-positive apoptotic cells and DAPI (blue)-stained nuclei in testicular sections from P60 mice. (**E**) Quantification of Casp3-positive cells from (**D**). Casp3-positive cells were counted in WT, *Cep152*^W105*/K897*^, and *Cep152*^Q32P/Q32P^ (*n* = 4, 5, 5 animals; 556, 550, 550 tubules, respectively). **** *p* < 0.0001. Scale bars; 20 μm (**A left**, **D**), 5 μm (**A right**).

To investigate the underlying mechanisms of testicular hypoplasia, we analyzed mitotic progression and apoptosis in spermatogonia. Immunostaining for phospho-histone H3 (pHH3) revealed a significant reduction in mitotic cells within the seminiferous tubules of both *Cep152*^W105*/K897*^ and *Cep152*^Q32P/Q32P^ mice at P10 (Figure 3A,B), a stage corresponding to the early establishment and functional maturation of seminiferous tubules, including the onset of spermatogonial differentiation, blood–testis barrier formation by Sertoli cells, and maturation of adult-like Leydig cells. Centrosome integrity analysis showed a drastic decrease in the proportion of bipolar spermatogonia with two γ-tubulin foci, alongside an increased the cells with a single γ-tubulin focus (Figure 3C), indicating defects in centrosome duplication. Consistent with these findings, activated Caspase-3 staining demonstrated significantly increased apoptosis of spermatogonia in both *Cep152* mutant models at P60 (Figure 3D,E). Interestingly, although both mutants exhibited elevated apoptosis, the increase was slightly more pronounced in *Cep152*^W105*/K897*^ mice (Figure 3E). This contrasts with the phenotype observed in the nervous system, where *Cep152*^Q32P/Q32P^ mice exhibit markedly more severe mitotic defects and apoptosis than *Cep152*^W105*/K897*^ mice [26].

### 3.3. Hematological Abnormalities in Cep152 Mutant Mice

**Figure 4:**
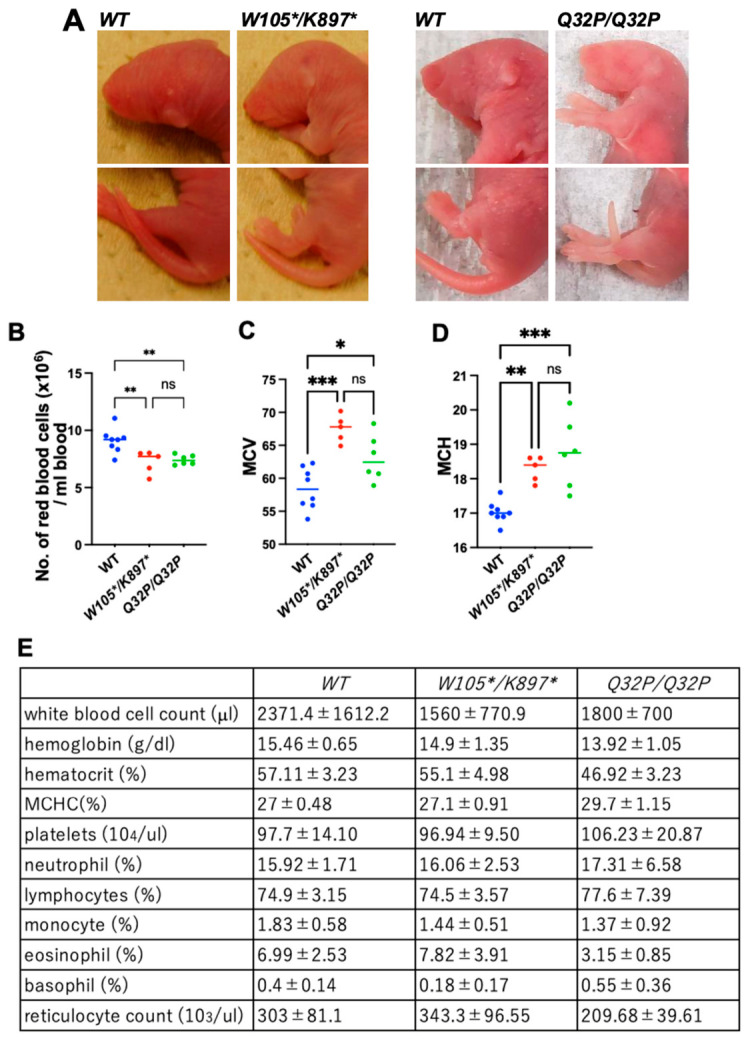
Anemia and impaired erythropoiesis in *Cep152* ^W105*/K897*^ and *Cep152*^Q32P/Q32P^ mice. (**A**) Representative images showing the heads and gluteal regions of WT, *Cep152*^W105*/K897*^, and *Cep152*^Q32P/Q32P^ mice at birth. (**B**) Quantification of red blood cell counts in WT, *Cep152*^W105*/K897*^, and *Cep152*^Q32P/Q32P^ mice at P60. (**C**,**D**) Quantification of mean corpuscular volume (MCV, (**C**)) and mean corpuscular hemoglobin (MCH, (**D**)) in mice of the indicated genotypes at P60. (**E**) Peripheral hemograms at P60. *n* = 8, 5, and 6 for WT, *Cep152*^W105*/K897*^, and *Cep152*^Q32P/Q32P^, respectively. * *p* < 0.05, ** *p* < 0.01, *** *p* < 0.001.

Both *Cep152*^W105*/K897*^ and *Cep152*^Q32P/Q32P^ mice typically presented with a pale surface body appearance at P0 (Figure 4A). Hematologic analysis revealed similarly reduced erythrocyte numbers in both mutant lines, accompanied by increased mean corpuscular volume (MCV) and mean corpuscular hemoglobin (MCH) levels (Figure 4B–D). These findings suggest impaired erythropoiesis, characterized by macrocytic anemia. In contrast, white blood cell and platelet counts were comparable to those in wild-type controls (Figure 4E), indicating that the mutant mice exhibit isolated erythroid defects without pancytopenia, a common feature of bone marrow failure. Consistently, immunohistochemical analysis of bone marrow from *Cep152*^W105*/K897*^ mice revealed no marked morphological abnormalities compared to wild-type littermates (Supplementary Figure S1). Notably, another SCKL mouse model, *Atr*-deficient mice, has been reported to exhibit pancytopenia, as observed in SCKL patients [28]. Together, these findings suggest that while *ATR* variants may lead to bone marrow failure, pathogenic *CEP152* variants primarily affect erythropoiesis without compromising overall hematopoiesis.

### 3.4. Enrichment in Gliogenesis-Related Genes Is Impaired in Cep152^W105*/K897*^ Mice

**Figure 5:**
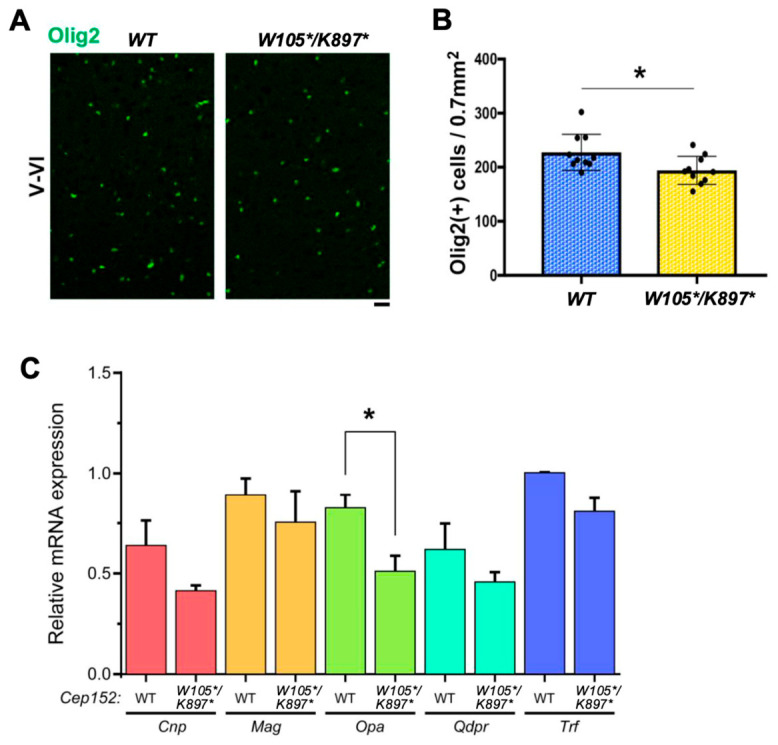
Impaired oligodendrocyte differentiation in *Cep152*^W105*/K897*^ mice. (**A**) Cortical slices (P60) were immunostained for Olig2. Representative images of cortical layer V/VI are shown. (**B**) Quantification of Olig2-positive cells in (**A**). The number of Olig2-positive cells per unit area (0.7 mm^2^) is shown as mean ± SD from 10 fields per mouse (*n* = 5 for each genotype). Statistical significance was determined by Welch’s *t*-test (* *p* < 0.05). (**C**) Quantitative reverse transcription PCR (RT-PCR) analysis of genes identified as downregulated in the cortices of *Cep152*^W105*/K897*^ mice based on RNA-seq data. Total RNA was extracted from the brains of 4 WT and 4 *Cep152*^W105*/K897*^ mice and subjected to quantitative RT-PCR. Relative mRNA expression levels were normalized to the WT value of *Trf* (set to 1.0). * *p* < 0.05. Gene abbreviations: *Cnp*; 2′,3′-Cyclic nucleotide 3′ phosphodiesterase; *Mag*, Myelin associated glycoprotein; *Opa*, Oligodendrocytic myelin paranodal and inner loop protein; *Qdpr*, Quinoid dihydropteridine reductase; *Trf*, Transferrin. Scale bar; 20 μm (**A**).

In our previous study, differential gene expression analysis of brains from adult *Cep152*^Q32P/Q32P^ mice revealed altered expression of genes related to synaptic formation and function, dendritic morphology, and axon development [26]. However, similar analysis of *Cep152*^W105*/K897*^ mice showed no individual genes reaching genome-wide significance among the 15,449 robustly expressed genes [26]. Despite the lack of genome-wide transcriptional changes, we found in immunohistochemical analysis a significant reduction in the number of Olig2-positive oligodendrocyte lineage cells in the cerebral cortex of *Cep152*^W105*/K897*^ mice (Figure 5A,B), suggesting possible alterations in oligodendrocyte development. Prompted by this observation in *Cep152*^W105*/K897*^ mice, we hypothesized that cell type-specific transcriptional changes might have been masked in the bulk RNA-seq data, and therefore quantified the expression of several oligodendrocyte-related genes (*Cnp*, *Mag*, *Opalin*, *Qdpr*, and *Trf*) in cortical tissues using quantitative RT-PCR. While all these genes exhibited a trend toward reduced expression in mutant mice, only *Opalin* showed a statistically significant decrease compared to wild-type controls (Figure 5C). Although exploratory in nature, these findings raise the possibility that CEP152 mutations may affect oligodendrocyte-related cellular processes. Together with the abnormalities observed in the blood and testis, these preliminary findings suggest that CEP152 dysfunction may affect multiple cell populations beyond neurons.

## 4. Discussion

In this study, we investigated the extra-neuronal consequences of pathogenic *CEP152* variants using two mouse models of SCKL, *Cep152*^W105*/K897*^ and *Cep152*^Q32P/Q32P^, which harbor patient-derived mutations. Previous work has predominantly focused on neurodevelopmental abnormalities in these models, particularly their roles in brain size reduction and cortical malformations [26]. Here, we extended the phenotypic analysis to include the testes and hematopoietic system. The analyses were performed at different developmental stages depending on the biological process examined. Testicular morphology was analyzed at both P10 and P60 to evaluate developmental progression and adult phenotypes, respectively. In contrast, analyses of spermatogonial progenitors and mitotic abnormalities were performed at P10, when proliferative activity in the seminiferous tubules is more prominent. Hematological analyses were conducted at P60 to assess stable systemic phenotypes in adult mice.

Our analyses revealed common cellular defects in proliferative spermatogonial progenitors, where both CEP152 variants impaired centrosome duplication, disrupted mitotic progression, and increased apoptosis. These findings suggest that CEP152 is required for maintaining genomic stability and viability in rapidly dividing cells beyond neural tissues, providing a mechanistic basis for the reproductive abnormalities observed in SCKL patients. Interestingly, despite differences in neurological severity, testicular and hematopoietic abnormalities were comparable between the two mutant models. This observation raises the possibility that tissue-specific factors or compensatory mechanisms modulate the phenotypic impact of CEP152 variants in a variant- and tissue-dependent manner. For example, differences in proliferative demand, developmental timing, or tolerance to mitotic stress among organs may influence the severity of cellular defects caused by CEP152 dysfunction. In addition, certain tissues may possess compensatory pathways that partially preserve centrosome function or cell viability despite impaired centriole duplication. Consistent with this possibility, we did not detect obvious histological abnormalities in several other highly proliferative tissues, including the femoral bone marrow, intestinal epithelium, and skin of *Cep152*^W105*/K897*^ mice (Supplementary Figure S1). These findings may further support the notion that the pathological consequences of CEP152 dysfunction are influenced by tissue-specific susceptibility and/or compensatory mechanisms.

An additional consideration is that the phenotypes observed in *Cep152*^W105*/K897*^ and *Cep152*^Q32P/Q32P^ mice may not simply reflect reduced CEP152 dosage. Complete loss of *Cep152* results in embryonic lethality (Hamada et al., unpublished observation), indicating that total absence of CEP152 function is incompatible with normal development. In contrast, the patient-derived variants analyzed here retain at least partial protein function, allowing survival while causing severe developmental abnormalities. Furthermore, our previous biochemical analyses suggested that these variants exert distinct effects on centrosomal function. CEP152-Q32P retained centrosomal localization despite impaired interaction with PLK4, whereas CEP152-W105* and CEP152-K897* showed marked protein instability or abnormal subcellular localization. Thus, these mutant proteins may disrupt centrosome organization or centriole assembly in a manner that differs from simple loss of function. Such residual but aberrant CEP152 activity may contribute to the pathological manifestations observed in the knock-in mice and could explain why variant-specific phenotypes do not necessarily correlate with the consequences of complete CEP152 deficiency.

Hematological assessment demonstrated signs of macrocytic anemia, a feature that is supposed to align with systemic manifestations in SCKL. However, in contrast to *Atr* mutant mice, which exhibit pancytopenia and severe bone marrow failure, *Cep152*^W105*/K897*^ and *Cep152*^Q32P/Q32P^ mice showed no significant reduction in white blood cells or platelets. These findings suggest that *CEP152* variants preferentially impair erythropoiesis rather than causing global hematopoietic failure, highlighting mechanistic differences among SCKL-associated genes.

Additionally, upon re-examining neural tissues, we observed a decrease in Olig2-positive cells in the cerebral cortex, along with reduced expression of the oligodendrocyte-related gene *Opalin* in *Cep152*^W105*/K897*^ mice. Although the effects on neurogenesis and neuronal architecture have been characterized previously, these findings raise the possibility that CEP152 dysfunction may influence oligodendrocyte-related cellular processes or white matter homeostasis. Further studies will be necessary to clarify the pathological significance of these observations.

## 5. Conclusions

The present study strongly suggests that pathogenic *CEP152* variants impair cellular proliferation in multiple tissues, contributing to systemic phenotypes characteristic of SCKL. Importantly, the observed phenotypic differences across tissues and between variants underscore the complexity of CEP152′s role in development and disease, providing insights into the pathophysiological mechanisms driving this multi-organ disorder.

## Data Availability

The data that support the findings of this study are available from the corresponding authors, upon reasonable request.

## Supplementary Materials

The following supporting information can be downloaded at: https://www.mdpi.com/article/10.3390/cells15131148/s1, Figure S1: Histological examination of highly proliferative tissues in Cep152^W105*/K897*^ mice. Representative hematoxylin and eosin (H&E)-stained sections of femoral bone marrow, intestinal epithelium, and skin from wild-type (WT) and Cep152^W105*/K897*^ mice at P60. Tissues were dissected, paraffin-embedded, sectioned at 4 μm, and stained with H&E. No obvious histological abnormalities were observed in the mutant tissues compared with WT controls. Images were acquired using a Keyence BZ-9000 microscope. Scale bars, 200 μm (*upper* panels) and 50 μm (*lower* panels).

## Abbreviations

The following abbreviations are used in this manuscript:

CEP152	Centrosomal protein 152
SCKL	Seckel syndrome
pHH3	phospho-histone H3

## References

[1] Bettencourt-Dias M., Glover D.M. (2007). Centrosome Biogenesis and Function: Centrosomics Brings New Understanding. Nat. Rev. Mol. Cell Biol..

[2] Conduit P.T., Wainman A., Raff J.W. (2015). Centrosome Function and Assembly in Animal Cells. Nat. Rev. Mol. Cell Biol..

[3] Takeda Y., Kuroki K., Chinen T., Kitagawa D. (2020). Centrosomal and Non-Centrosomal Functions Emerged through Eliminating Centrosomes. Cell Struct. Funct..

[4] Ganem N.J., Godinho S.A., Pellman D. (2009). A Mechanism Linking Extra Centrosomes to Chromosomal Instability. Nature.

[5] Basto R., Brunk K., Vinadogrova T., Peel N., Franz A., Khodjakov A., Raff J.W. (2008). Centrosome Amplification Can Initiate Tumorigenesis in Flies. Cell.

[6] Nigg E.A. (2002). Centrosome Aberrations: Cause or Consequence of Cancer Progression?. Nat. Rev. Cancer.

[7] Bettencourt-Dias M., Hildebrandt F., Pellman D., Woods G., Godinho S.A. (2011). Centrosomes and Cilia in Human Disease. Trends Genet..

[8] Davis E.E., Katsanis N. (2012). The Ciliopathies: A Transitional Model into Systems Biology of Human Genetic Disease. Curr. Opin. Genet. Dev..

[9] Nogales-Cadenas R., Carmona-Saez P., Vazquez M., Vicente C., Yang X., Tirado F., Carazo J.M., Pascual-Montano A. (2009). GeneCodis: Interpreting Gene Lists through Enrichment Analysis and Integration of Diverse Biological Information. Nucleic Acids Res..

[10] Andersen J.S., Wilkinson C.J., Mayor T., Mortensen P., Nigg E.A., Mann M. (2003). Proteomic Characterization of the Human Centrosome by Protein Correlation Profiling. Nature.

[11] Varmark H., Llamazares S., Rebollo E., Lange B., Reina J., Schwarz H., Gonzalez C. (2007). Asterless Is a Centriolar Protein Required for Centrosome Function and Embryo Development in Drosophila. Curr. Biol..

[12] Blachon S., Gopalakrishnan J., Omori Y., Polyanovsky A., Church A., Nicastro D., Malicki J., Avidor-Reiss T. (2008). Drosophila Asterless and Vertebrate Cep152 Are Orthologs Essential for Centriole Duplication. Genetics.

[13] Dzhindzhev N.S., Yu Q.D., Weiskopf K., Tzolovsky G., Cunha-Ferreira I., Riparbelli M., Rodrigues-Martins A., Bettencourt-Dias M., Callaini G., Glover D.M. (2010). Asterless Is a Scaffold for the Onset of Centriole Assembly. Nature.

[14] Cizmecioglu O., Arnold M., Bahtz R., Settele F., Ehret L., Haselmann-Weiß U., Antony C., Hoffmann I. (2010). Cep152 Acts as a Scaffold for Recruitment of Plk4 and CPAP to the Centrosome. J. Cell Biol..

[15] Fırat-Karalar E.N., Stearns T. (2014). The Centriole Duplication Cycle. Philos. Trans. R. Soc. Lond. B Biol. Sci..

[16] Varadarajan R., Rusan N.M. (2018). Bridging Centrioles and PCM in Proper Space and Time. Essays Biochem..

[17] Kalay E., Yigit G., Aslan Y., Brown K.E., Pohl E., Bicknell L.S., Kayserili H., Li Y., Tüysüz B., Nürnberg G. (2011). CEP152 Is a Genome Maintenance Protein Disrupted in Seckel Syndrome. Nat. Genet..

[18] Kodani A., Yu T.W., Johnson J.R., Jayaraman D., Johnson T.L., Al-Gazali L., Sztriha L., Partlow J.N., Kim H., Krup A.L. (2015). Centriolar Satellites Assemble Centrosomal Microcephaly Proteins to Recruit CDK2 and Promote Centriole Duplication. eLife.

[19] Sir J.-H., Barr A.R., Nicholas A.K., Carvalho O.P., Khurshid M., Sossick A., Reichelt S., D’Santos C., Woods C.G., Gergely F. (2011). A Primary Microcephaly Protein Complex Forms a Ring around Parental Centrioles. Nat. Genet..

[20] Firat-Karalar E.N., Rauniyar N., Yates J.R., Stearns T. (2014). Proximity Interactions among Centrosome Components Identify Regulators of Centriole Duplication. Curr. Biol..

[21] Brown N.J., Marjanović M., Lüders J., Stracker T.H., Costanzo V. (2013). Cep63 and Cep152 Cooperate to Ensure Centriole Duplication. PLoS ONE.

[22] Park S.-Y., Park J.-E., Kim T.-S., Kim J.H., Kwak M.-J., Ku B., Tian L., Murugan R.N., Ahn M., Komiya S. (2014). Molecular Basis for Unidirectional Scaffold Switching of Human Plk4 in Centriole Biogenesis. Nat. Struct. Mol. Biol..

[23] Sonnen K.F., Gabryjonczyk A.-M., Anselm E., Stierhof Y.-D., Nigg E.A. (2013). Human Cep192 and Cep152 Cooperate in Plk4 Recruitment and Centriole Duplication. J. Cell Sci..

[24] Shanske A., Caride D.G., Menasse-Palmer L., Bogdanow A., Marion R.W. (1997). Central Nervous System Anomalies in Seckel Syndrome: Report of a New Family and Review of the Literature. Am. J. Med. Genet..

[25] Griffith E., Walker S., Martin C.-A., Vagnarelli P., Stiff T., Vernay B., Sanna N.A., Saggar A., Hamel B., Earnshaw W.C. (2008). Mutations in Pericentrin Cause Seckel Syndrome with Defective ATR-Dependent DNA Damage Signaling. Nat. Genet..

[26] Hamada N., AlAbdi L., Uehara T., Sasikarn L., Nishijo T., Suliman-Lavie R., Hashem O.M., Alfadhel M., Alhefdhi S., Tabarki B. (2026). Distinct pathophysiological mechanisms of *CEP152* variants in microcephaly and brain abnormalities. EMBO Mol. Med..

[27] Kawai S., Takagi Y., Kaneko S., Kurosawa T. (2011). Effect of Three Types of Mixed Anesthetic Agents Alternate to Ketamine in Mice. Exp. Anim..

[28] Murga M., Bunting S., Montaña M.F., Soria R., Mulero F., Cañamero M., Lee Y., McKinnon P.J., Nussenzweig A., Fernandez-Capetillo O. (2009). A Mouse Model of ATR-Seckel Shows Embryonic Replicative Stress and Accelerated Aging. Nat. Genet..

