# Supplementary material for: Extra-Neurological Characterization of Seckel Syndrome-Model Mice Harboring CEP152 Variants
Source: Cells. 2026 Jun 24;15(13):1148. doi: 10.3390/cells15131148 (PMC13359663; doi:10.3390/cells15131148)
Supplement: Supplementary file 1 [file cells-15-01148-s001.zip › cells-4330200-supplementary.pdf]

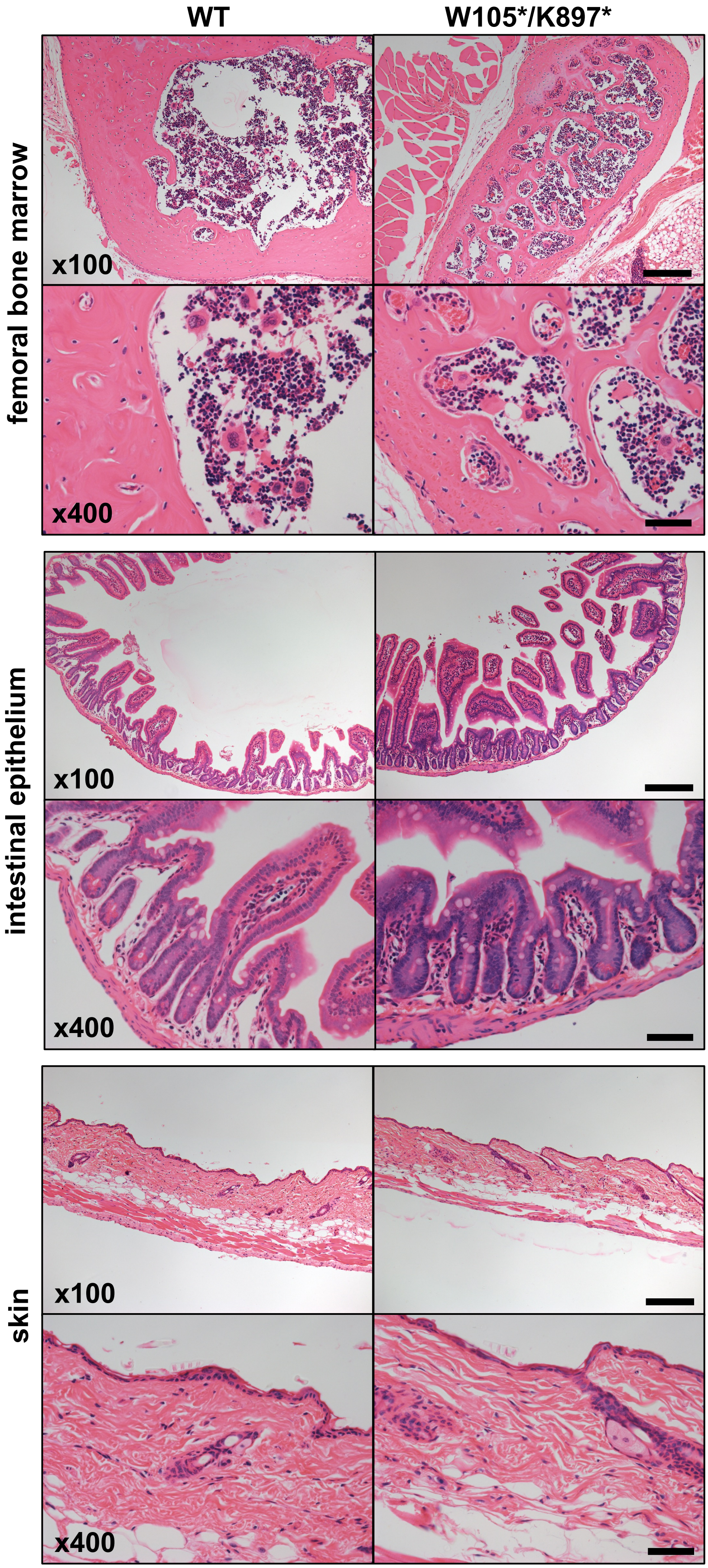

Supplementary Figure S1. Histological examination of highly proliferative tissues in Cep152$^{W105*/K897*}$ mice. Representative hematoxylin and eosin (H&E)-stained sections of femoral bone marrow, intestinal epithelium, and skin from wild-type (WT) and Cep152$^{W105*/K897*}$ mice at P60. Tissues were dissected, paraffin-embedded, sectioned at 4 µm, and stained with H&E. No obvious histological abnormalities were observed in the mutant tissues compared with WT controls. Images were acquired using a Keyence BZ-9000 microscope. Scale bars, 200 µm (upper panels) and 50 µm (lower panels).
